# Can consumers accurately identify loot boxes and loot box receipts?

**DOI:** 10.1098/rsos.261484

**Published:** 2026-10-21

**Authors:** Matt B. Hall, Lauren C. Hall, James D. Sauer, Aaron Drummond

**Affiliations:** ^1^ School of Psychology, Massey University, Palmerston North, New Zealand; ^2^ School of Psychological Sciences, University of Tasmania, Hobart, Australia

**Keywords:** loot boxes, gambling, consumer protections, parents, video games

## Abstract

Loot boxes are randomized rewards in video games. Meta-analyses suggest relationships between loot box spending and problem gambling symptomatology, and early longitudinal evidence suggests loot box purchasers migrate into conventional gambling at higher rates than non-purchasers. Regulatory responses vary across jurisdictions, and even in jurisdictions that have banned loot boxes, evidence suggests their presence in games remains common. Consequently, consumers are often responsible for regulating their own, and their children’s, loot box purchasing. To effectively achieve this, consumers must first be able to identify loot boxes. Here, we investigated consumer accuracy in identifying critical features of loot boxes. Participants were tasked to identify basic facts about, pictures of, and receipts generated by loot boxes. Parents and non-gamers, compared with non-parents and gamers, respectively, were poorer at identifying loot boxes. Our results suggest that parents and non-gamers may be less equipped to make informed loot box purchasing decisions compared with non-parents/non-guardians and gamers, respectively. This may pose risks for parents and unaware incipient gamers. These are the first findings providing objective evidence about the relative ability of consumers to identify, and thus self-regulate purchases of, loot boxes and suggest that parents and non-gamers may require support to ensure informed purchasing decisions.

## 1. Introduction

Loot boxes are digital containers of randomized rewards available for purchase within some video games [1]. The randomized nature of loot boxes has raised concerns that they psychologically [1] and legally approximate conventional gambling [2]. Concordantly, there is a highly replicable association between problem gambling symptoms and loot box spending [3–8]. Emerging longitudinal [9,10], prospective [11] and retrospective evidence [12] suggests loot box purchasers migrate into conventional gambling at higher rates than non-purchasers. Players at greatest risk of gambling harm typically spend the most on loot boxes across virtually every income category [13,14]. Removing loot boxes specifically reduces expenditure from players with problem gambling symptoms [15]. Together, these findings raise concerns about vulnerable players, including underage players and those at risk of gambling harm, engaging with loot boxes. However, most current consumer protection efforts rely upon individuals regulating their own loot box spending. To do this, consumers—and their parents—must first be able to identify loot boxes. Here, we investigated this issue in a sample of adult (age = 18+) gamers and parents.

Regulatory responses towards loot boxes have varied widely between jurisdictions. Although some countries (e.g. Belgium) have implemented bans, such bans have not been particularly effective at eliminating loot boxes from games [16]. Moreover, loot boxes remain unregulated in many jurisdictions. Where regulatory efforts have been applied, they are frequently focused upon the provision of consumer information for informed decision-making. For instance, the Pan European Game Information (PEGI) rating system that provides content labels across 40 countries, requires games with loot boxes to provide a ‘Contains Paid Random Items’ content descriptor [17]. This label appears presently poorly understood by consumers [17], and the label implementation is lacking uniformity, particularly for online-only releases [18].

The laissez-faire nature of regulatory frameworks for loot boxes in many jurisdictions places a substantial burden on consumers seeking to make informed decisions about whether to, and how to, engage with loot boxes. Where labelling is absent, or inadequate [18], players (or parents/guardians of players) wanting to make an informed choice about whether to engage with loot boxes must first be able to recognize the presence of loot boxes in games without this being specifically enumerated to them. Even where labelling is present and accurate, it is a necessary precondition for a player or parent who wishes themselves or their child to engage with a game but not its embedded loot boxes that they can accurately recognize those loot boxes. Given the wide array of aesthetic characteristics that a loot box might possess [19], this task is not straightforward.

Two groups might be reasonably expected to struggle to identify loot boxes: parents and non-gamers. Each is interesting for different theoretical and applied reasons. Parents are perhaps the most obvious group to examine from an applied perspective, because many parents are involved in deciding which games their children are allowed to play and spend money on. Herein, by parents, we refer to all individuals who are parents or guardians of children and thus have legal responsibility for a child.

Evidence suggests that at least some parents are active in attempting to regulate their children’s loot box engagement. Gong & Rodda [20] found that parents commonly used strategies such as removing access to loot boxes (e.g. by removing the gaming console or computer) and uninstalling games containing loot boxes. Parents also frequently attempted to educate children about loot box mechanisms and the value of money to highlight the consequences of engaging with games of chance [20]. Yet, if parents are to be successful in this, parents themselves must first be able to reliably identify loot boxes.

There is good theoretical reason to expect that parents may not be well-placed to identify loot boxes in video games. Parents are often time-poor [21,22], creating challenges for deep learning and engagement with their children’s hobbies (e.g. video games). In turn, this lack of familiarity may reduce parents’ ability to identify problematic features. This is exacerbated by the solitary nature of many games. Concordantly, Dong *et al.* [23] surveyed parents and found that many did not know what a loot box was. This suggests that parents may presently be ill-equipped to identify the presence of loot boxes *in situ*. This invites further examination of parental capacity to recognize and regulate loot box engagement for their children.

The importance of parental ability to identify loot boxes and their digital traces (e.g. digital receipts) is highlighted by multiple news reports of children spending substantial amounts of money on loot boxes without their parents’ knowledge. One teenager reportedly spent $7625.88 on in-game currency that was then spent to purchase loot boxes [24]. Many digital gaming stores save credit card details by default for the ease of future purchases. Although convenient, this may create opportunities for children to enact purchases without re-entering credit card details. Loot box purchases generate a receipt sent to the email address associated with the purchasing account. Therefore, many parents’ first knowledge of loot boxes purchased in this way may be a receipt from a transaction their child has made. It is therefore important that parents can successfully identify loot box receipts to quickly enact regulation strategies.

The theoretical reasons underpinning the potential for poorer understanding for non-gamers are perhaps obvious (i.e. non-gamers have less involvement in, knowledge about and familiarity with games and their features). However, the importance of understanding their ability to recognize loot box features from an applied perspective may be less intuitive. This group is important to consider for at least two reasons. First, not all parents are gamers, and thus it is possible that parents who are also non-gamers might be cumulatively poorer at identifying loot box features compared with gamer peers. Second, not all gamers begin video-gaming in youth. Taking up the hobby later in life might render these players less able to identify these features, potentially leading to inadvertent or unintentional engagement. In other words, examining the understanding of non-gamers gives us insight into the potential baseline understanding about loot boxes for nascent gamers. Thus, we believe non-gamers are an important group to consider herein.

Here, we employed a signal detection theory approach [25] to examine the degree to which adult (age = 18+) parents and non-gamers were poorer than their non-parent and gamer peers at (i) identifying the characteristics of loot boxes in a general knowledge test; (ii) detecting the presence of loot boxes based on real screenshots of loot boxes from video games; and (iii) identifying digital receipts for the purchase of loot boxes in video games. We expected both parents and non-gamers to have lower knowledge of the gaming environment. Specifically, we predicted that on a loot box general knowledge test, on a test identifying images of loot boxes in games, and in a test identifying loot box purchasing receipts:

— (H1) Parents and guardians will have lower accuracy, discriminability (*d*′) and area under curve (AUC) in a receiver operating characteristic (ROC) curve analysis than non-parents and non-guardians on a basic loot box knowledge test.
— (H2) Non-video gamers will have lower accuracy, *d*′ and AUC in a ROC curve analysis than gamers on a basic loot box knowledge test.
— (H3) Guardianship status will interact with video gameplay status such that gamers without children will have the highest *d*′ scores and AUC scores in identifying loot boxes and loot box receipts compared with all other groups.

Note, these hypotheses have been streamlined from the pre-registration at the request of a reviewer. The revised wording condenses the hypotheses without changing their meaning. The original hypotheses are available in the pre-registration document.

## 2. Material and methods

### 2.1. Pre-registration

Our hypothesis, sampling plan, stopping rule, exclusion criteria, measures and statistical models were pre-registered with OSF (see https://osf.io/2sbez/overview).

### 2.2. Design

The present study employed a cross-sectional, between-subjects, quasi-experimental design. Participants were members of natural groups (e.g. parents/non-parents, gamers/non-gamers) and completed a series of true/false questions about loot boxes.

### 2.3. Participants

Participants were 600 (278 male; 302 female, 13 non-binary, 2 other and 1 prefer-not-to-say) Australian (62), Aotearoa New Zealand (30) and USA (504) adults (18+ years). Thirty-one participants were parent non-gamers, 181 parent gamers, 45 non-parent non-gamers and 338 non-parent gamers. One participant did not report their parental status and was not included in the analyses. The mean age of participants was 33.68 (s.d. = 11.6). Three participants did not provide their age. Following the application of our pre-registered exclusion criteria, five participants were excluded (four for mischievous responding and one for not answering the parental status question). Thus, the final sample size was 595 participants.

### 2.4. Measures

#### 2.4.1. Parental status

Participants were asked: ‘Are you a parent or a guardian of children?’ (‘Yes/No’).

#### 2.4.2. Gamer status

Participants were asked: ‘How often do you play video games?’ (‘never, monthly, weekly, most days, daily’). We dichotomized this into two groups—participants who do not play video games (i.e. ‘never’) and participants who play video games at least occasionally (i.e. all other answers).

#### 2.4.3. Dependent measures

The full scales are available on OSF here: https://osf.io/5sud2/files/xhq28. The dependent measures consisted of *d*′ and AUC scores on a general loot box knowledge test, identification of loot boxes and identification of loot box receipts. For all measures, *d*′ was calculated as *d*′ = *z* (hit rate) – *z* (false alarm rate). A flattening constant of 0.5/1.0 was applied in the computation of *d*′ scores to allow for the calculation of *d′* if participants got zero answers correct (which would otherwise result in *d*′ being unable to be calculated). Higher *d*′ scores indicated greater performance on this task, whereas a zero indicates no ability to discern true from false information. AUC was computed using the geometric method described in Green & Swets [25]. The analysis of *d*′ and AUC scores is more appropriate than raw accuracy scores for these kinds of data because raw accuracy has the potential to conflate *d*′ and response bias, and does not account for task difficulty. For instance, consider two hypothetical participants: participant A and participant B. Participant A consistently correctly gives higher confidence scores to target stimuli than non-target stimuli, but does not rate any stimulus above the midpoint of the scale. By contrast, participant B does not consistently give higher confidence to target over non-target stimuli, and also does not rate any stimuli about the midpoint of the scale. In this case, participant A is consistently rating target stimuli higher than non-targets (i.e. demonstrating that they can discriminate between target and non-target stimuli) and is only slightly underconfident and/or the task was slightly too difficult for participant A to answer confidently. In comparison, participant B is consistently reporting higher confidence for non-target stimuli (i.e. failing to effectively discriminate target from non-target stimuli). Under raw accuracy rates, both would be considered equivalent (i.e. consistently incorrect). In comparison, under AUC analyses, participant A would be given a higher AUC score owing to their tendency to rate target stimuli higher than non-target stimuli, allowing for greater nuance in the analysis and accounting for response bias and task difficulty effects. For more detail, see Green & Swets [25].

#### 2.4.4. Loot box general knowledge questions

Participants’ comprehension of loot boxes was assessed using 10 (5 true and 5 false) items such as ‘A loot box delivers a random reward to the player’ (True), and ‘Players can choose what rewards they receive from loot boxes’ (False). Participants responded to these items on a six-point Likert-type scale from 1 (Certain that this statement is false), to 6 (Certain that this statement is true). Responses of 1–3 were classified as false, and 4–6 were classified as true. Data were converted to *d*′ indices. The maximum possible score on this test was *d*′ = 2.77.

#### 2.4.5. Loot box image identification questions

This measure consisted of a series of 10 items (5 true and 5 false) containing an image with or without a loot box (see https://osf.io/5sud2/files/xhq28 for full scale). Participants provided responses to ‘How certain are you that this screenshot contains a loot box?’ on the six-point scale outlined above. As noted in the procedure below, participants were provided with a definition of a loot box before answering these questions. Data were converted to *d*′ indices. The maximum possible score on this test was *d*′ = 2.77.

#### 2.4.6. Loot box receipt image identification questions

This measure also consisted of a series of 10 items (5 true and 5 false) containing an image of a receipt with or without the presence of a loot box on the receipt. Participants provided responses to the question ‘How certain are you that this screenshot contains a loot box receipt?’ on the same six-point scale as above. Data were converted to *d*′ indices. The maximum possible score on this test was *d*′ = 2.75.

#### 2.4.7. Additional measures

Participants completed a series of questions assessing their loot box familiarity. This was assessed with questions such as ‘How familiar are you with in-game purchases?’, ‘Are you familiar with what a loot box is?’ and ‘Have you ever purchased a loot box for real-life money?’. Participants responded to these items on a seven-point Likert-type scale from 1 (not at all familiar) to 7 (extremely familiar). We did not pre-register any analyses incorporating these familiarity questions, and they were collected as exploratory variables only.

#### 2.4.8. Procedure

Data were gathered through an online survey advertised on Prolific and hosted on Qualtrics. Participants were presented with the 10 questions measuring general loot box knowledge. The set ended with an additional attention check question. Participants were then presented with the following definition of a Loot box: ‘“Loot box” is a broad term that defines a specific kind of transaction in video games. Loot boxes are rewards within video games, sometimes purchased for real money or using in-game currency that costs money. Loot boxes are any digital container, card pack, crate or case that contains an unknown reward at the time of purchase or being earned.’

Following this, participants answered the 10 loot box image questions, one image at a time. The order that each image appeared in was randomized for each participant. After the completion of the 10 loot box image questions, participants were then presented with the 10-question set of loot box receipt images. Again, stimulus materials were presented one image at a time, each of which either contained a loot box receipt or not, and the order was randomized. Finally, participants were then asked what gender they identified with, their age, and what country they lived in.

### 2.5. Data analysis

Participant responses to the factual questions were transformed into *d*′ scores. Higher *d*′ scores index greater ability to discriminate between true and false claims (or presence and absence of loot boxes/receipts), with a score of 0 indicating no ability to distinguish between items. AUC scores similarly index discrimination ability. AUC scores range from 0 to 1, with higher scores indicating better discrimination, and 0.5 indicating chance performance. Scores of less than 0.5 indicate a tendency to believe false over true items. Bayesian ANOVAs were conducted from the *d*′ and AUC scores using JAMOVI (v 2.5.5). Bayes factors are interpreted as the ratio of the likelihood of the data occurring under the alternative hypothesis to the likelihood of the data accruing under the null hypothesis. Table 1 presents guidelines for Bayes factor interpretation (adapted from [26]).

**Table 1.** Guidelines for the relative strength of evidence indexed by Bayes factors.

evidence category		
Bayes factor	BF10 or BF^+^	BF01 or BF^–^
>100	extreme evidence for H1	extreme evidence for H0
30–100	very strong evidence for H1	very strong evidence for H0
10–30	strong evidence for H1	strong evidence for H0
3–10	moderate evidence for H1	moderate evidence for H0
1–3	anecdotal evidence for H1	anecdotal evidence for H0
1	no evidence	no evidence

*Note:* Adapted from Lee & Wagenmakers [26].

## 3. Results

Our data are openly available here: https://osf.io/5sud2/overview.

### 3.1. Loot box general knowledge questions

A one-sample Bayesian *t*-test against the maximum possible score on this task (*d*′ = 2.77) showed that participants’ average *d*′ score (*M* = 1.62, s.d. = 0.91) on this task was below ceiling by a large amount, BF = 4.41 × 10^122^, Cohen’s *d* = 1.27. A Bayesian ANOVA yielded extreme evidence in favour of the model showing two independent main effects of parental status and gamer status on *d*′, BF_10_ = 4.76 × 10^6^. As predicted, parents (*M* = 1.44, s.d. = 0.95) had poorer *d*′ than non-parents (*M* = 1.72, s.d. = 0.87), BF_inclusion_ = 24.67, *d* = 0.31, a small difference. Similarly, non-gamers (*M* = 1.10, s.d. = 0.97) had moderately poorer *d*′ than gamers (*M* = 1.69, s.d. = 0.87), BF_inclusion_ = 7.64 × 10^4^, *d* = 0.64. Including the interaction term weakened the BF value by a factor of 4.99, providing moderate evidence against an interaction (BF_inclusion_ = 0.77).

The analysis of AUC scores confirmed this pattern, with the strongest evidence being for the presence of two main effects, BF_10_ = 4.56 × 10^6^. Again, as predicted, parents (*M* = 0.76, s.d. = 0.14) had poorer *d*′ than non-parents (*M* = 0.79, s.d. = 0.12), a small difference, BF_inclusion_ = 3.64, *d* = 0.23. Non-gamers (*M* = 0.71, s.d. = 0.13) had moderately poorer *d*′ than gamers (*M* = 0.79, s.d. = 0.12), BF_inclusion_ = 5.93 × 10^5^, *d* = 0.64. Again, including the interaction term weakened the BF value by a factor of 3.48, providing moderate evidence against an interaction (BF_inclusion_ = 0.91). Together, these analyses provide extreme evidence that parents (cf. non-parents) were slightly less accurate in their general knowledge about loot boxes (H1), and gamers (cf. non-gamers) were moderately more accurate in their general knowledge about loot boxes (H2).

### 3.2. Loot box image identification

Figure 1 shows *d′* scores for loot box image questions for parents, non-parents, gamers and non-gamers. A one-sample Bayesian *t*-test against the maximum possible score on this task (*d*′ = 2.77) showed that participants’ average *d*′ score (*M* = 1.49, s.d. = 0.84) on this task was below ceiling by a large amount, BF = 3.84 × 10^147^, Cohen’s *d* = 1.41. A Bayesian ANOVA revealed definitive evidence for the presence of main effects of parental and gamer status on *d′*, BF_10_ = 1.65 × 10^9^. As predicted, parents were less accurate in identifying the presence of loot boxes than non-parents (BF_inclusion_ = 103.18), and gamers were more accurate in identifying the presence of loot boxes than non-gamers (BF_inclusion_ = 6.68 × 10^6^). Including the interaction weakened the BF value by a factor of 4.24 (BF_inclusion_ = 0.88), providing moderate evidence against including the interaction term.

**Figure 1.**
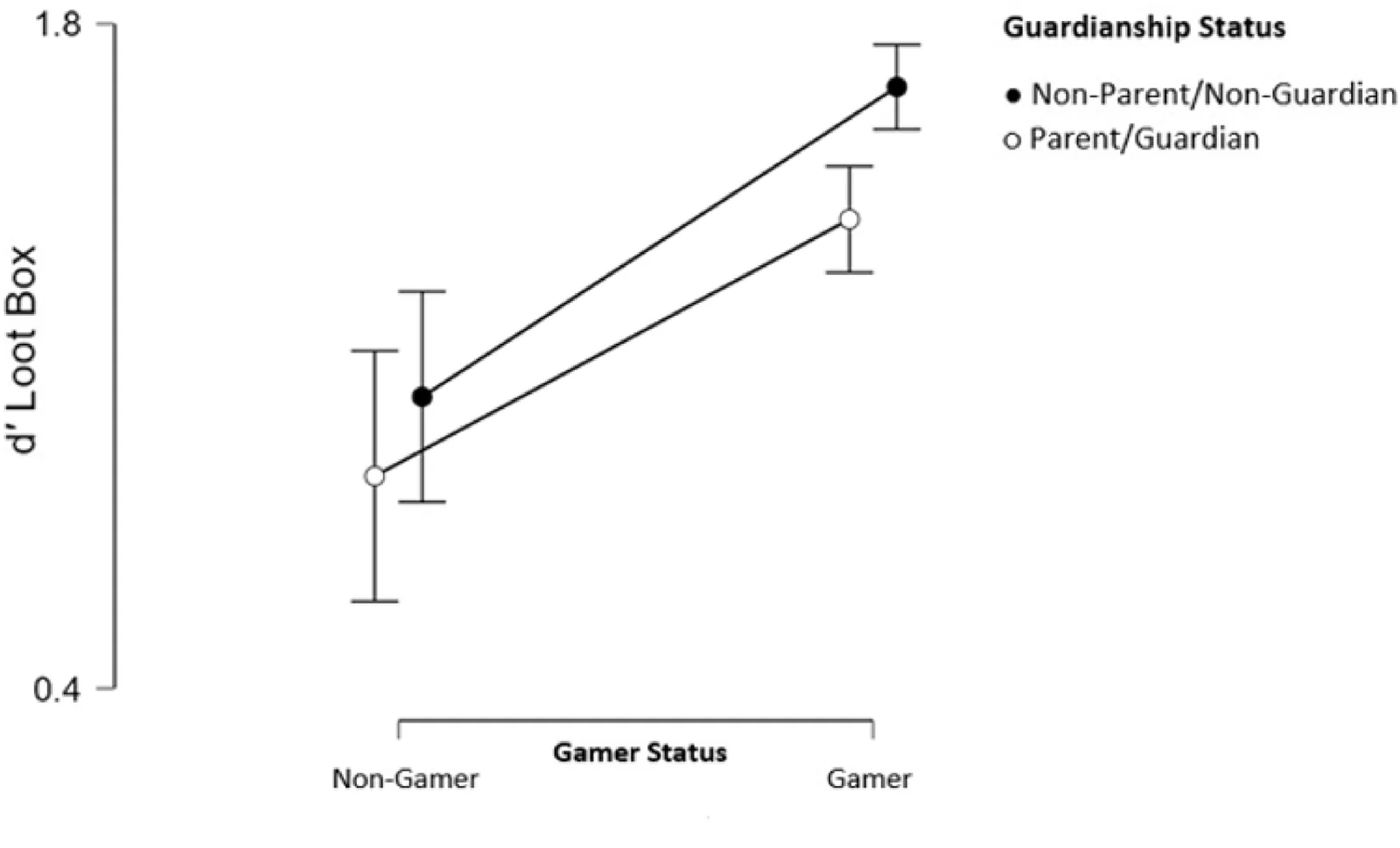
Discrimination scores for true/false loot box images. As hypothesized, parents performed poorer in discriminating between images containing loot boxes and images not containing loot boxes than non-parents. Non-gamers also performed worse in *d*′ than gamers did. The results indicate that being a parent or a non-gamer is associated with poorer performance in discriminating loot box images than being a non-parent or a gamer, respectively. Note that no evidence for an interaction between parental status and gamer status was observed, providing evidence against the hypothesis that parental status and gamer status would interact (H3). Error bars represent 95% credibility intervals.

The corresponding models with parental status and gamer status as predictors and AUC as an outcome measure revealed similar findings. The strongest evidence was for a model incorporating the two main effects, but no interaction BF_10_ = 5.19 × 10^8^. As predicted, parents were less accurate in identifying the presence of loot boxes (*M* = 0.72, s.d. = 0.13) than non-parents (*M* = 0.77, s.d. = 0.13), a small-to-moderate difference, BF_inclusion_ = 1031.51, *d* = 0.38. Similarly, gamers were moderately more accurate in identifying loot boxes (*M* = 0.77, s.d. = 0.13) than non-gamers (*M* = 0.68, s.d. = 0.12), BF_inclusion_ = 2.21 × 10^5^, *d* = 0.72. Including the interaction weakened the effect by a factor of 3.90, providing moderate evidence against an interaction (BF_inclusion_ = 1.02). Taken together, these findings provided evidence in support of Hypotheses 1 and 2, and partial evidence against H3 (i.e. there was no evidence for an interaction between parental and gamer statuses in the identification of loot boxes).

### 3.3. Loot box receipt identification

Figure 2 shows *d*’ scores for loot box receipt image questions for parents, non-parents, gamers and non-gamers. A one-sample Bayesian *t*-test against the maximum possible score on this task (*d*′ = 2.77) showed that participants’ average *d*′ score (*M* = 0.97, s.d. = 0.80) on this task was below ceiling by a large amount, BF = 3.28 × 10^228^, Cohen’s *d* = 2.22. A Bayesian ANOVA revealed extreme evidence in support of both parental and gamer status being associated with *d*’ on identifying loot box receipts, BF_10_ = 142.91. Again, as predicted, parents were less accurate than non-parents (BF_inclusion_ = 3.03), and non-gamers were less accurate than gamers (BF_inclusion_ = 44.48) at identifying loot box receipts. As in previous analyses, including the interaction weakened the effect (BF_10_ = 88.82), indicating evidence against an interaction (BF_inclusion_ = 1.99). These results also provide further evidence against the hypothesis that parental status and gamer status would interact (H3).

**Figure 2.**
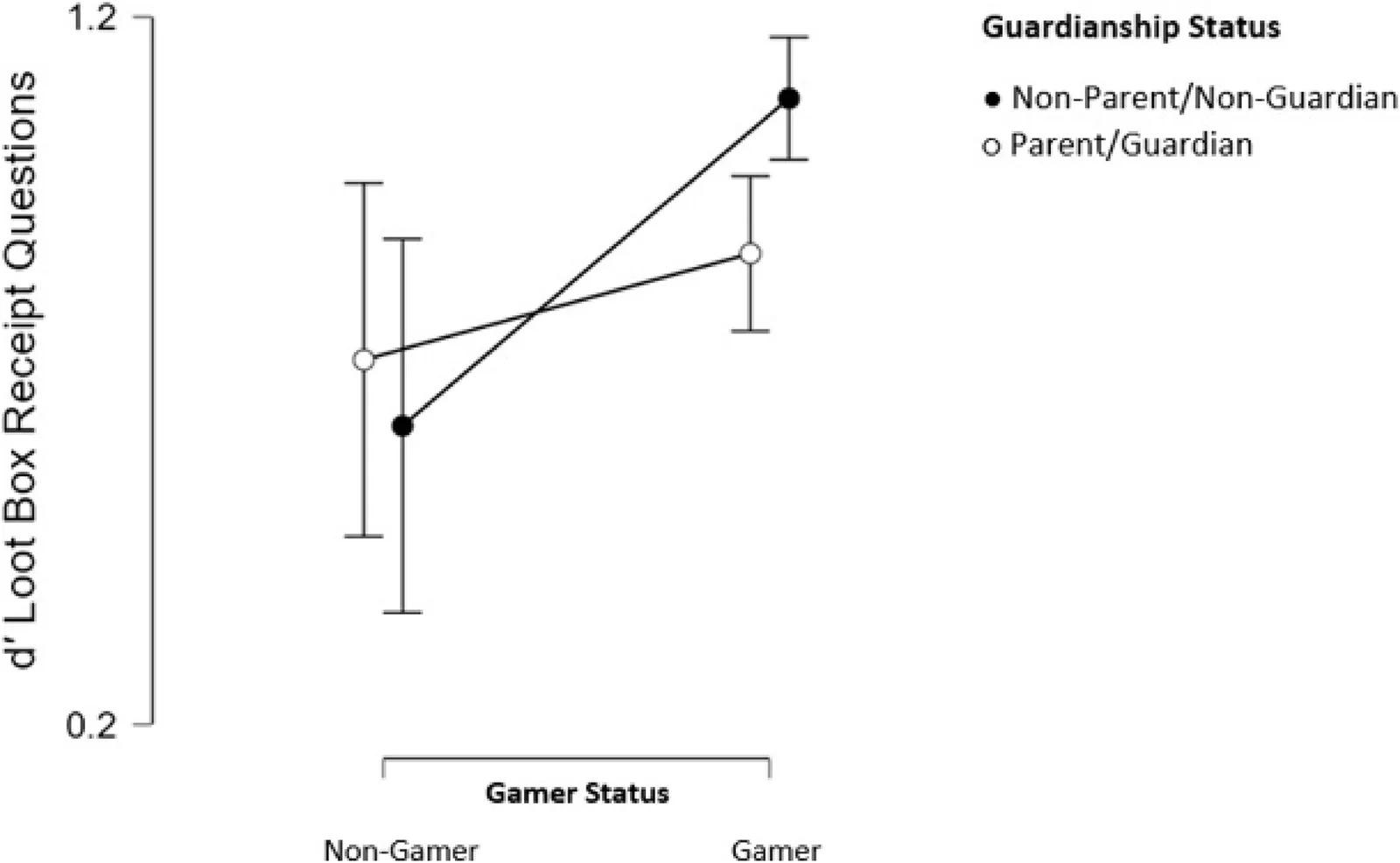
Discrimination scores for true/false loot box receipt images. As hypothesized, parents performed poorer in discriminating between images containing loot box receipts and images not containing loot box receipts than non-parents. Non-gamers also performed worse in *d*′ than gamers did. The results indicate that being a parent or non-gamer is associated with poorer performance in discriminating between loot box receipt images than being a non-parent or a gamer, respectively. No evidence for an interaction between parental status and gamer status was observed, providing evidence against H3 that parental status and gamer status would interact. Error bars represent 95% credibility intervals.

The analysis of AUC scores for loot box receipts was similar, though the evidence provided by the Bayes factors was stronger. There was extreme evidence for the presence of main effects of parental and gamer status, BF_10_ = 2.59 × 10^5^. As expected, parents were less accurate in identifying loot box receipts (*M* = 0.66, s.d. = 0.12) than non-parents (*M* = 0.70, s.d. = 0.13), a small-to-moderate difference, BF_inclusion_ = 38.89, *d* = 0.32. Similarly, gamers were moderately more accurate in identifying the presence of loot boxes (*M* = 0.69, s.d. = 0.13) than non-gamers (*M* = 0.62, s.d. = 0.12), BF_inclusion_ = 2938.34, *d* = 0.56. Including the interaction weakened the evidence for an effect by a factor of 3.57, providing moderate evidence against the inclusion of an interaction, BF_inclusion_ = 1.17.

## 4. Discussion

This study examined how accurately participants could identify loot boxes in terms of their characteristics, their visual presentation and their digital traces in the form of receipts. Parents and non-gamers performed more poorly at identifying all three facets of loot boxes compared with their non-parent and gamer peers. In all cases, participants were performing well below ceiling levels, suggesting that improvement in the ability to detect loot boxes and their digital receipts is needed across all consumer groups if these groups are to make informed decisions about their own loot box engagement, and the engagement of young people in their care.

For consumers to make informed decisions about whether to interact with loot boxes, it is a necessary precondition that parents and gamers can recognize loot boxes, their features and their receipts. Ideally, people should be able to recognize a loot box every time it is presented to them, to aid decisions about whether to engage. Neither of these situations appears true: parents are poorer than non-parents at identifying loot boxes, and overall, performance was objectively well below perfect. In all three *d*′ tests, effect sizes as indexed by Cohen’s *d* showed that participants were scoring below the maximum possible score by more than a full standard deviation. Scores were particularly low for the identification of loot box receipts that were over two standard deviations below the maximum possible score on average. Together, these findings suggest that consumers, especially non-gamers and parents, have difficulty recognizing loot boxes. Without being able to consistently and accurately identify a loot box, parents may struggle to enact the kinds of individualistic consumer protection afforded by current consumer protection policies (e.g. warning labels). Parents are also less able than non-parents to identify past purchases of loot boxes from their digital receipts. This suggests that when children purchase loot boxes without explicit permission, parents may not recognize this spending to curtail it. This is especially problematic if parents allow spending on non-randomized items but would prefer their children not engage with loot boxes.

Non-gamers, too, were poorer at the identification of loot boxes in games. Many non-gamers will not encounter loot boxes. However, parents who do not game may nonetheless need to make informed decisions on behalf of the young people in their care. If they are less able to identify loot boxes, they are less well-equipped to make these decisions. Furthermore, some non-gamers may begin gaming and then encounter the mechanisms without realizing it. This could be particularly problematic if they are substituting gaming for a gambling activity they are attempting to reduce or cease. Future research should examine whether individuals at risk of gambling harm with little experience of modern video games are migrating into the hobby, and whether this creates a unique risk to their recovery and continued non-participation in gambling.

Importantly, there was no interaction between parental and gamer status on loot box recognition. Holding both statuses simultaneously was not associated with multiplicative decrements in performance—that is, each status is associated with decreased performance, but holding both is not associated with additional multiplicative reduced performance beyond their individual impacts. Superficially, this result appears problematic, as for parents to make informed decisions, even parents with gaming experience are at reduced performance compared with their non-gaming parental peers. However, if the gamers outperform non-gamers on this task owing to greater experience, then exposure to games for parents might plausibly improve task accuracy. Further research is needed to ascertain whether targeted education interventions to improve knowledge may help mitigate the reduced ability for parents to recognize loot boxes.

Critically, our results are qualified by the quasi-experimental methods employed. As we did not manipulate gamer or parental status, we cannot conclude these factors are causal in determining performance on recognizing loot boxes. It may be that third variables, such as interest in gaming, are partly or wholly responsible for the associations. Although our data do not allow us to conclusively rule it out, we think reverse causality is unlikely: that is, having a better ability to identify loot boxes seems intuitively unlikely to cause a lack of children. In any case, this is a rare instance where applied relevance is more important than causal directionality. Irrespective of why parents and non-gamers have poorer performance in identifying loot boxes, that these groups demonstrably *have* poorer ability to identify loot boxes places them at a disadvantage when making informed decisions about whether they (or their children) should engage with these mechanisms. If individualistic consumer-centred approaches to loot boxes are to be the prevailing policy response, such differences, and the relatively middling recognition performance overall, pose substantive challenges to ensuring that consumers are making well-informed purchasing decisions and, consequently, to the likely efficacy of this consumer-protection approach. The combination of low consumer awareness, poorer parental recognition and the general trend towards consumer awareness approaches (e.g. warning labels) for loot boxes leads to two necessary corollaries. First, public ability to identify loot boxes appears to currently be low, especially for parents and non-gamers. This may limit the efficacy of the self-regulatory approaches towards loot boxes taken by industry and adopted in many countries [27]. Second, further public education campaigns to ensure that consumers are adequately placed to identify these features in games and their resultant digital traces (e.g. receipts) to accommodate informed consumer decisions are probably required.

The small sample sizes evident in the two non-gamer groups are a second limitation of the present project. Moreover, it is possible that some demographic factors such as age and gender may influence results, either acting as confounding variables or creating undue noise in the data. Future research should aim to replicate the present findings with a larger group of non-gamers to better understand the impact of gaming experience upon the ability to identify the presence and digital trace data associated with loot boxes in video games, and explore whether the inclusion of demographic controls improves model fit. Future research may also seek to understand whether the degree of experience gamers have playing games (e.g. the frequency they play games and the breadth of their gaming experience) may be associated with incremental improvements in performance on loot box detection tasks. Relatedly, future research should investigate the degree to which children are able to identify the presence and associated risks of loot boxes to allow for informed decisions about whether to engage in concert with parental oversight. Future research investigating the degree to which children of parents who play video games also play video games, and how this impacts their ability to identify and experience of, loot boxes in games, may be a fruitful avenue of inquiry.

This study sought to understand how well consumers are able to identify the presence of loot boxes in video games. The results demonstrate that consumers are well below ceiling levels (and only slightly above chance performance) in their ability to detect the presence of loot boxes in games and purchases of loot boxes through digital trace receipts. Furthermore, our results suggest that parents and non-gamers are both less able to identify loot boxes and their receipts than their non-parent and gamer peers, respectively. Being both a parent and a non-gamer did not appear to be associated with multiplicatively lower performance, suggesting that public awareness campaigns may be effective at educating parents about loot boxes, though further clear labelling of loot boxes within games and receipts would also be beneficial. Efforts to improve consumer discernibility to the presence of loot boxes in games, and legibility of loot box receipts are essential to ensuring consumers are able to make well-informed purchasing decisions for themselves and their children.

## Data Availability

Data, complete with analyses, are available at OSF [28].
